# Probiotics and Paraprobiotics: Effects on Microbiota-Gut-Brain Axis and Their Consequent Potential in Neuropsychiatric Therapy

**DOI:** 10.1007/s12602-024-10214-6

**Published:** 2024-01-31

**Authors:** Samriti Balaji Mudaliar, Sumith Sundara Poojary, Alevoor Srinivas Bharath Prasad, Nirmal Mazumder

**Affiliations:** 1 Department of Public Health & Genomics, Manipal School of Life Sciences, Manipal Academy of Higher Education, Manipal, Karnataka 576104 India; 2 Department of Biophysics, Manipal School of Life Sciences, Manipal Academy of Higher Education, Manipal, Karnataka 576104 India

**Keywords:** Paraprobiotics, Probiotics, Gut-brain axis, Neuropsychiatry, Gut microbiome, Immunomodulation

## Abstract

Neuropsychiatric disorders are clinical conditions that affect cognitive function and emotional stability, often resulting from damage or disease in the central nervous system (CNS). These disorders are a worldwide concern, impacting approximately 12.5% of the global population. The gut microbiota has been linked to neurological development and function, implicating its involvement in neuropsychiatric conditions. Due to their interaction with gut microbial communities, probiotics offer a natural alternative to traditional treatments such as therapeutic drugs and interventions for alleviating neuropsychiatric symptoms. Introduced by Metchnikoff in the early 1900s, probiotics are live microorganisms that provide various health benefits, including improved digestion, enhanced sleep quality, and reduced mental problems. However, concerns about their safety, particularly in immunocompromised patients, warrant further investigation; this has led to the concept of “paraprobiotics”, inactivated forms of beneficial microorganisms that offer a safer alternative. This review begins by exploring different methods of inactivation, each targeting specific cellular components like DNA or proteins. The choice of inactivation method is crucial, as the health benefits may vary depending on the conditions employed for inactivation. The subsequent sections focus on the potential mechanisms of action and specific applications of probiotics and paraprobiotics in neuropsychiatric therapy. Probiotics and paraprobiotics interact with gut microbes, modulating the gut microbial composition and alleviating gut dysbiosis. The resulting neuropsychiatric benefits primarily stem from the gut-brain axis, a bidirectional communication channel involving various pathways discussed in the review. While further research is needed, probiotics and paraprobiotics are promising therapeutic agents for the management of neuropsychiatric disorders.

## Introduction

Neuropsychiatry refers to the scientific and medical approach toward conditions that include both neurological and psychological manifestations. It seeks to integrate neuroscience and psychiatry in the assessment and subsequent treatment of such conditions [1]. Neuropsychiatric disorders encompass a range of diseases that affect both the brain and mental health. They are highly prevalent globally, impacting individuals of all ages and backgrounds. As per reports from the World Health Organization (WHO), an estimated 12.5% of the global population is afflicted by neuropsychiatric disorders such as anxiety, bipolar disorder, major depressive disorder (MDD), attention-deficit/hyperactivity disorder (ADHD), and autism spectrum disorder (ASD). These disorders, especially those associated with erratic mood changes, have been identified as a significant cause of suicide [2]. It is evident that neuropsychiatric disorders are a prevalent and significant concern in the global health arena, and they demand attention and allocation of resources for their mitigation. Studies have linked the gut microbiota to neurological development and brain function through the gut-brain axis (GBA), suggesting that changes in the gut microbial populations could influence the development of neuropsychiatric symptoms [3]. Given this connection, beneficial microbes offer a natural therapeutic alternative to conventional drugs for treating and alleviating the symptoms of neuropsychiatric conditions. The term “bacteria” or “microbes” is often associated with the stigma that they are inherently harmful. However, this perception is not entirely accurate, as they can be both beneficial and detrimental depending on the context. While some microbes can cause infections and diseases, others play essential roles in maintaining human health and the environment. Probiotics represent these beneficial viable microbes that provide health benefits when consumed in appropriate amounts [4, 5].

Ilya Metchnikoff, a scientist from Russia, is credited with putting forth the idea of using live microorganisms to enhance human well-being in the early 1900s. He noted that individuals from Bulgaria who consumed fermented dairy products lived longer and experienced fewer illnesses, and he attributed these positive outcomes to microbes present in their fermented dairy. This concept laid the groundwork for the modern understanding of probiotics and the crucial role of the gut microbiome in health. Today, several microbes, mainly bacteria and yeasts, are considered probiotics, such as *Lactobacillus reuteri*, *Lactobacillus rhamnosus*, *Lactobacillus casei*, *Bifidobacterium bifidum*, and *Saccharomyces boulardii*.

However, the health benefits they provide are still being investigated by ongoing scientific studies [6, 7]. Furthermore, the exact means through which they optimize the gut microbiome and provide health benefits are still not completely understood but evidence suggests the involvement of multiple factors such as amensalism via the release of antimicrobials, stimulating the secretion of mucus to protect the gut lining, contending with invading pathogenic microbes for adhesion sites, stabilizing gut barrier function, and inducing immunological responses, while the psycho-emotional benefits are mediated by the gut-brain axis (GBA) [8, 9].

Probiotics exist in a variety of fermented, dairy, and non-dairy food products such as yogurt, kefir, sauerkraut, kimchi, and miso which can be included in correct portions in the daily diet; alternatively, they can also be taken separately as supplements in the form of tablets or capsules in appropriate doses. The global probiotics market is anticipated to compound at a rate of 7.5% annually till 2030, starting with a value of 58.17 billion US dollars in 2021 [10].

Despite the potential benefits, several concerns have been associated with probiotic use. Due to the widespread and inappropriate use of antibiotics, several probiotics have been shown to exhibit resistance to widely used antibiotics. Thus, the possibility of horizontal transfer of these resistance genes to pathogens or the transfer of virulence genes from pathogens to probiotics is a pressing issue [11, 12]. Furthermore, there have been reports of infections such as bacteremia, fungemia, sepsis, and endocarditis in immunodeficient patients and patients having underlying disorders such as AIDS and ulcerative colitis[13–15]. Moreover, metabolic effects such as D-lactic acidosis also have been observed [16]. Hence, safety assessments of probiotics, particularly concerning specific population subsets such as immunocompromised patients, are required before they can be commercially produced.

A new concept of paraprobiotics, also known as non-viable, dead, inactivated, or ghost probiotics, has been introduced to represent inactivated microbes that provide various health benefits, including immunomodulatory, anti-inflammatory, antioxidant, and anti-hypertensive effects. Paraprobiotics can be defined as non-viable microbial cells, as well as cellular components, microbial fractions, and bacterial lysates, that improve the health of the host through interactions with the gut microbiome when administered in sufficient quantity. They generally comprise peptidoglycans, polysaccharides from the microbial cell walls, cell surface proteins, and teichoic acid [17]. Substantial evidence, both in vitro and in vivo, has shown that the beneficial effects of paraprobiotics are comparable to those exhibited by probiotics, despite the former consisting of only dead or inactivated cells that lack the ability to proliferate and colonize in the gut microbiota, unlike live probiotics that can interact with gut microbes of the host thereby regulating the composition of the gut microbiota. This seemingly counterintuitive phenomenon has been termed the “probiotic paradox” [18]. While additional research is required to elucidate the mechanism of action of paraprobiotics, studies have reported certain methods by which they exert health benefits, such as host interactions mediated by dead cell components leading to an immune response, and production of beneficial microbial metabolites including peptides and short-chain fatty acids (SCFAs) [18, 19].

**Fig. 1:**
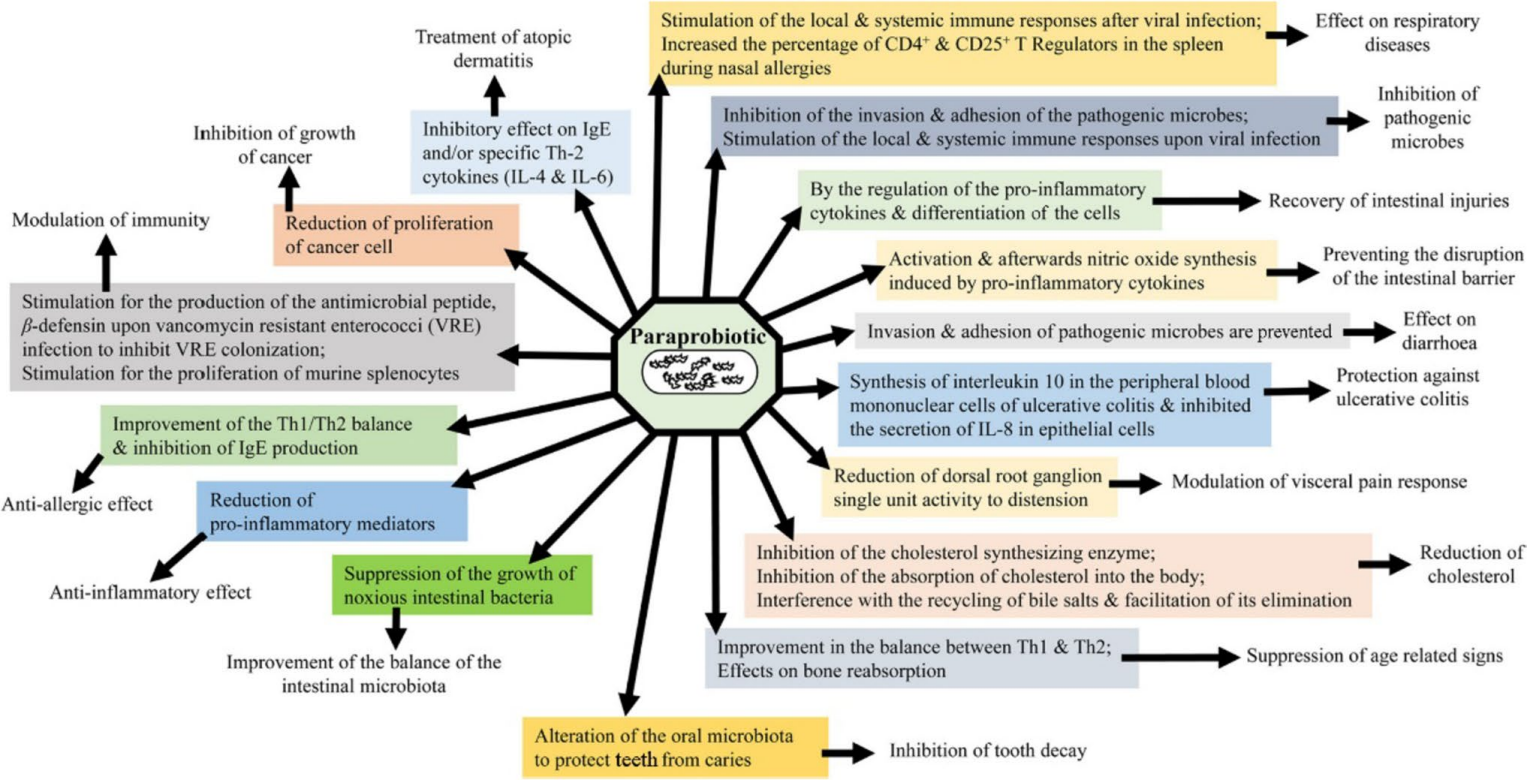
Health benefits exerted by paraprobiotics [21]

The health advantages associated with both probiotics and paraprobiotics are multifaceted. These benefits include improved sleep quality, alleviation of symptoms associated with irritable bowel syndrome (IBS), and potential anti-cancerous effects, as illustrated in Fig. 1. However, this review primarily focuses on the neuropsychiatric benefits of probiotics and paraprobiotics. Paraprobiotics present several compelling advantages when compared to probiotics, particularly regarding their safety and potential to positively impact host health. As previously explained, probiotics, which contain live bacteria could be problematic for patients with underlying disorders and carry several safety issues. In contrast, paraprobiotics do not carry such risks since they are rendered inviable prior to administration. This characteristic enhances their likelihood of receiving regulatory approval for use as supplements or functional ingredients in food products. Another noteworthy benefit of paraprobiotics is their extended shelf life and lack of interaction with the other constituents of food products. They also maintain structural and functional stability across a wide temperature range. This simplifies their management and transportation within the food industry. Furthermore, paraprobiotics display bioactivity even when incorporated into non-dairy food matrices. This is significant because non-dairy substrates are typically challenging environments for the survival of traditional probiotics. Moreover, the capacity of paraprobiotics to thrive in such matrices has the potential to diversify the functional food market by allowing their integration into a broader spectrum of products [20].

This review introduces the potential therapeutic effects of probiotics and paraprobiotics in the management of neuropsychiatric disorders like anxiety and MDD in light of their effect on the gut microbiota which, in turn, affects neural functioning through the GBA. The main objectives of this review are to explain the concept of probiotics and paraprobiotics, to describe the microbiota-gut-brain axis and its significance, and finally, to highlight the potential of probiotics and paraprobiotics in treating neuropsychiatric problems. The first section of the review focuses on the different inactivation methods employed for the conversion of probiotics into paraprobiotics, namely, thermal treatment, supercritical carbon dioxide (SC-CO_2_) technology, high pressure, ohmic heating, sonication, ionizing radiation (IR), pulsed electric field (PEF), and ultraviolet (UV) rays. The process involved in each inactivation method has been briefly explained followed by details of its mechanism of action and examples of its application. In the subsequent section, the focus shifts to the GBA and its connection to neuropsychiatric disorders. The signaling pathways and regulatory constituents involved in the GBA, viz., the vagus nerve, immunological activity, inflammatory reflex and neuroinflammation, microbial metabolites, and neuroactive compounds are explored. The influence of each of these elements on the development of neuropsychiatric disorders like ASD is also discussed in this section. Lastly, the final section delves into the applications of probiotics and paraprobiotics in alleviating the symptoms of neuropsychiatric problems. The results of preclinical studies and clinical trials are delineated in order to provide the readers with an overview of the current status of the ongoing research on probiotics and paraprobiotics in neuropsychiatric therapy.

## Inactivation Methods

**Fig. 2:**
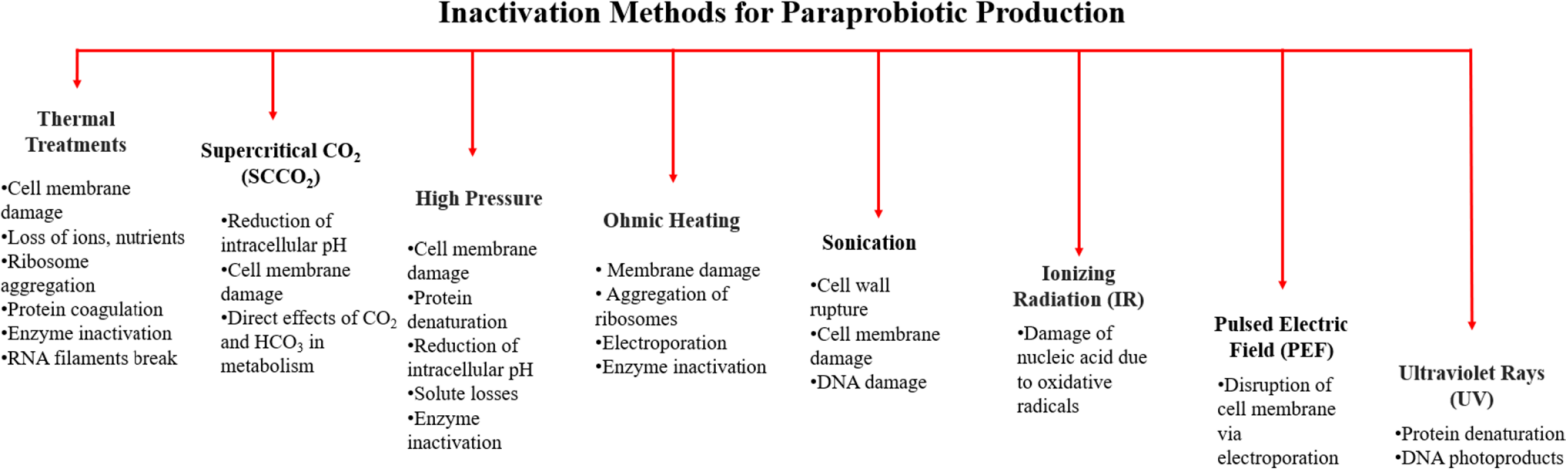
Common methods for the production of paraprobiotics [19, 21]

Paraprobiotics can be obtained from live probiotics through chemical means using acids and formalin or through physical processes such as thermal treatment, ohmic heating, and ionizing radiation (IR), as highlighted in Fig. 2, with each method having different mechanisms that alter their structural properties and may have contrasting health effects. Hence, monitoring the impact of various inactivation methods on the structural properties of the bacteria as well as the quantitative and qualitative maintenance of probiotic properties by each of these methods is extremely crucial [20, 22].

### Thermal Treatment

Thermal treatment is highly conventional and the most widely used process in the production of paraprobiotics since it is well-developed and requires low investment costs. It involves the exposure of probiotic species to high temperatures, thereby inducing cellular damage, as explained in Table 1, consequently making them non-viable.

**Table 1:** Sites of damage due to exposure to heat

**Site**	**Damage**	**References**
**Cell wall/outer membrane**	The outer membrane (OM) of Gram-negative cells is one of the structures affected by heat. Loss of lipopolysaccharide and vesicles. Morphological and structural changes or blebbing have also been reported.	[23, 30]
**Peptidoglycan wall**	The chelation of magnesium ions in the cell wall had an impact on vital metabolic processes within the cell. It was shown that exposing *Lactobacillus bulgaricus* cells to a temperature of 64 °C caused damage to their cell walls since it increased their susceptibility to penicillin.	[23, 30, 31]
**Cytoplasmic/inner membrane**	Cells become leaky which eventually leads to cell death. Loss of respiration activity, osmotic homeostasis, and pH homeostasis. The loss of ions, UV-absorbing substances, and other cytoplasmic materials has also been reported in various species.	[31–35]
**Ribosomes and RNA**	RNA degradation. Ribosome degradation was also observed which depended on the concentration of magnesium ions in the medium which increases ribosomal resistance toward heat.	[23, 30]
**DNA**	Single-strand breaks (SSB) or double-strand breaks (DSB) are introduced.	[23, 30, 36]

Thermal treatments can be classified into two categories based on the extent of heat applied and the goal—pasteurization and sterilization. Pasteurization, named after Louis Pasteur, involves the use of mild temperatures (< 100 °C). It may further involve inactivation by heating to higher temperatures (72 °C) for around 15 s or about 63 °C for 30 min; while the former is an example of high-temperature short time (HTST) pasteurization, the latter uses the low-temperature long time (LTLT) method. Though pasteurization may not be suitable for inactivation of spore-forming probiotics such as *Bacillus coagulans*, these spore-forming microbes can be inactivated by other methods that have been subsequently discussed. Sterilization involves the use of higher temperatures (> 100 °C in most cases) for a short time to render the probiotics non-viable or inactive [19, 23–25].

The thermal resistance of probiotic species is one of the parameters that need to be considered during this treatment. The *D*-values and *Z*-values are employed to measure how resistant the microbial species are to heat or thermal treatments. The *D*-value, also known as decimal reduction time, is defined as the time required at a specific temperature to reduce the cell viability to 10% while the *Z*-value is the temperature necessary to bring about a tenfold reduction in the *D*-value [19, 25].

Microorganisms display diverse responses to temperature changes, and these responses are shaped by the differences in their mechanisms for resisting heat. The ability to withstand heat is a critical aspect for microorganisms, as it determines their ability to survive and thrive in varying temperature environments. Certain strains of probiotics such as *L. plantarum* can accumulate osmoprotectants like glycine betaine or trehalose. These substances serve a protective role by stabilizing proteins and cellular structures, safeguarding cells from damage caused by elevated temperatures, ensuring the maintenance of proper protein folding, preserving cellular membranes, and preventing water loss, thereby enhancing the resilience of probiotic cells in the face of heat stress [26, 27]. Others produce heat shock proteins (HSPs) such as GroEL and GroES when subjected to high temperatures [28, 29]. Thus, heat resistance mechanisms differ based on the specific strains employed due to differences in the genetic constitution, physiology, and form, i.e., vegetative or sporous, and environmental factors like growth medium, temperature, and pH, among others, which need to be examined individually for assessing the response of probiotics to heat treatments and thereby optimizing the inactivation protocol.

Therefore, understanding the temperature ranges is crucial for the development of efficient thermal treatment protocols and the dosage may vary accordingly. Standardization is achieved by subjecting microorganisms to diverse temperatures over varying time periods and evaluating their viability. The resulting data aids in the determination of heat resistance characteristics specific to the microorganisms under study, forming the basis for their heat resistance profile. Other methods to determine thermal inactivation include the thermal death time (TDT) tube method and the submerged-coil heating apparatus [24]. Studies comparing the dosage and duration of the inactivation treatment against the health benefits of the inactivated paraprobiotics are necessary to develop an appropriate inactivation protocol.

### *S*upercritical Carbon Dioxide (SC-CO_2_) Technology

The principle behind utilizing the supercritical carbon dioxide (SC-CO_2_) technology to inactivate microbes lies in leveraging the unique properties of supercritical CO_2_. Carbon dioxide (CO_2_) has a critical point value of 31 °C and 7.38 MPa. In this state, CO_2_ exhibits both gas- and liquid-like properties, enabling enhanced solvating power and diffusion capabilities along with low viscosity [37, 38]. Optimizing key parameters such as temperature, pressure, and exposure time is necessary to achieve effective microbial inactivation.

SC-CO_2_ can penetrate the cell membrane of microorganisms due to its low viscosity and high diffusivity. This penetration disrupts membrane integrity, resulting in increased permeability and leakage of intracellular components. When SC-CO_2_ dissolves in water or aqueous solutions, it forms carbonic acid, which leads to a decrease in pH. The acidic environment can disrupt microbial cellular processes and compromise their survival. Furthermore, SC-CO_2_ can extract lipids and other essential cellular components from microbial cells. The supercritical fluid’s solvating power allows it to dissolve and remove lipids, proteins, and other crucial components necessary for microbial viability [39, 40].

### High Pressure

The use of high pressure to inactivate microbes is another emergent technique that finds application in paraprobiotic production. In this technique, microorganisms are suspended in a fluid medium like water that allows for pressure transmission and then subjected to high hydrostatic pressure ranging from 30 to 350 MPa in a high-pressure homogenizer [41].

**Fig. 3:**
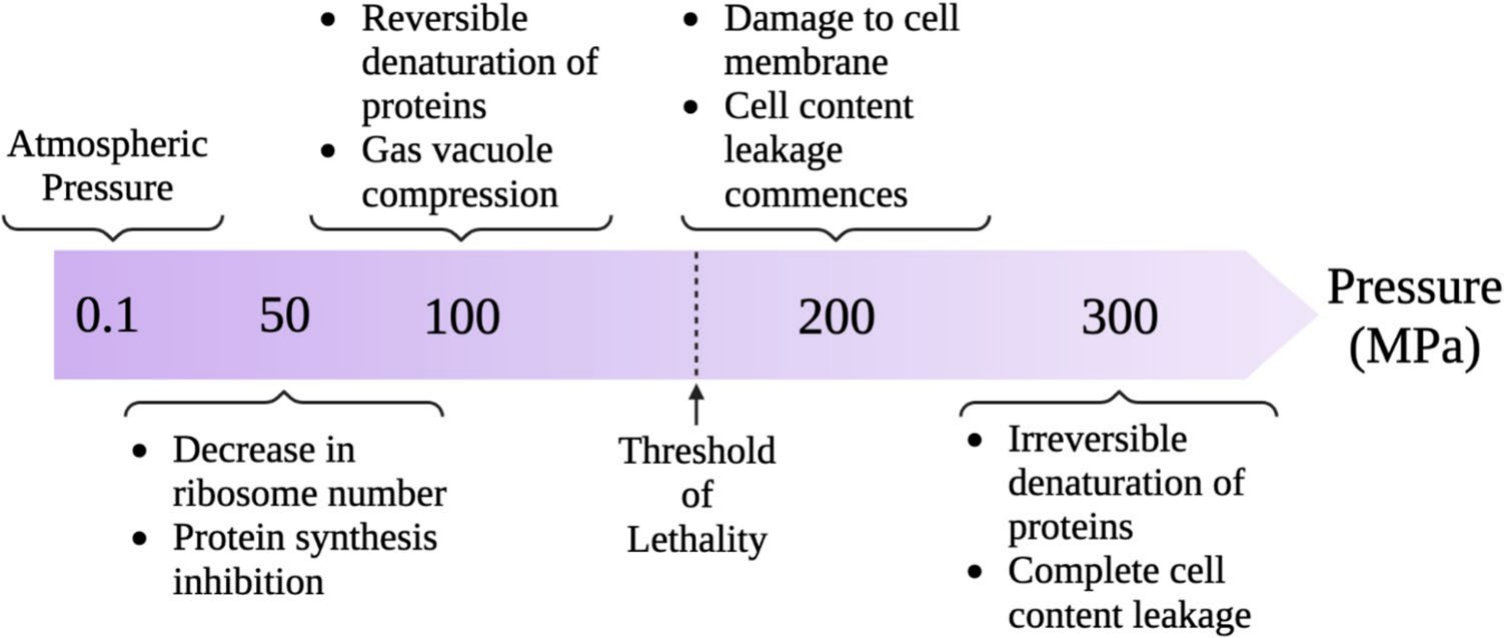
Cellular alterations caused by high pressure [42]

The range of pressure employed as well as the treatment duration are both very critical in this technique. As demonstrated in Fig. 3, the effect on the microbe varies for different pressure intervals. At a pressure of 50 MPa, protein synthesis is inhibited along with a decrease in ribosome number, and upon a one-fold increase in pressure to around 100 MPa, vacuoles get compressed, and proteins are reversibly denatured. Further increase in pressure to 200 MPa crosses the threshold of lethality leading to cell membrane damage which causes subsequent leakage of cell contents. Finally, the proteins undergo irreversible denaturation accompanied by the complete leakage of cell contents at around 300 MPa [42]. Deadly impact to the cell is seen upon irreversible disturbances in the transport system coupled to the membrane at pressures greater than 400 MPa. As a result, depending on the treatment, intensity, and process duration, varying impacts on cell integrity will be produced [43].

Microbes that have been inactivated by high pressure do not lose their probiotic properties since specific disrupted cell fractions are recognized by members of the immune system, thus eliciting a proinflammatory response. Additionally, certain probiotics continue to secrete microbial metabolites even after the pressure exceeds the threshold of lethality [18]. Though it is apparent that the increase in pressure is directly proportional to the rate of inactivation, equipment design limitations and the required extent of cell inactivation must also be considered. In order to decrease the duration and pressure, this technique can be used in combination with other inactivation methods such as thermal treatment [44].

### Ohmic Heating

**Fig. 4:**
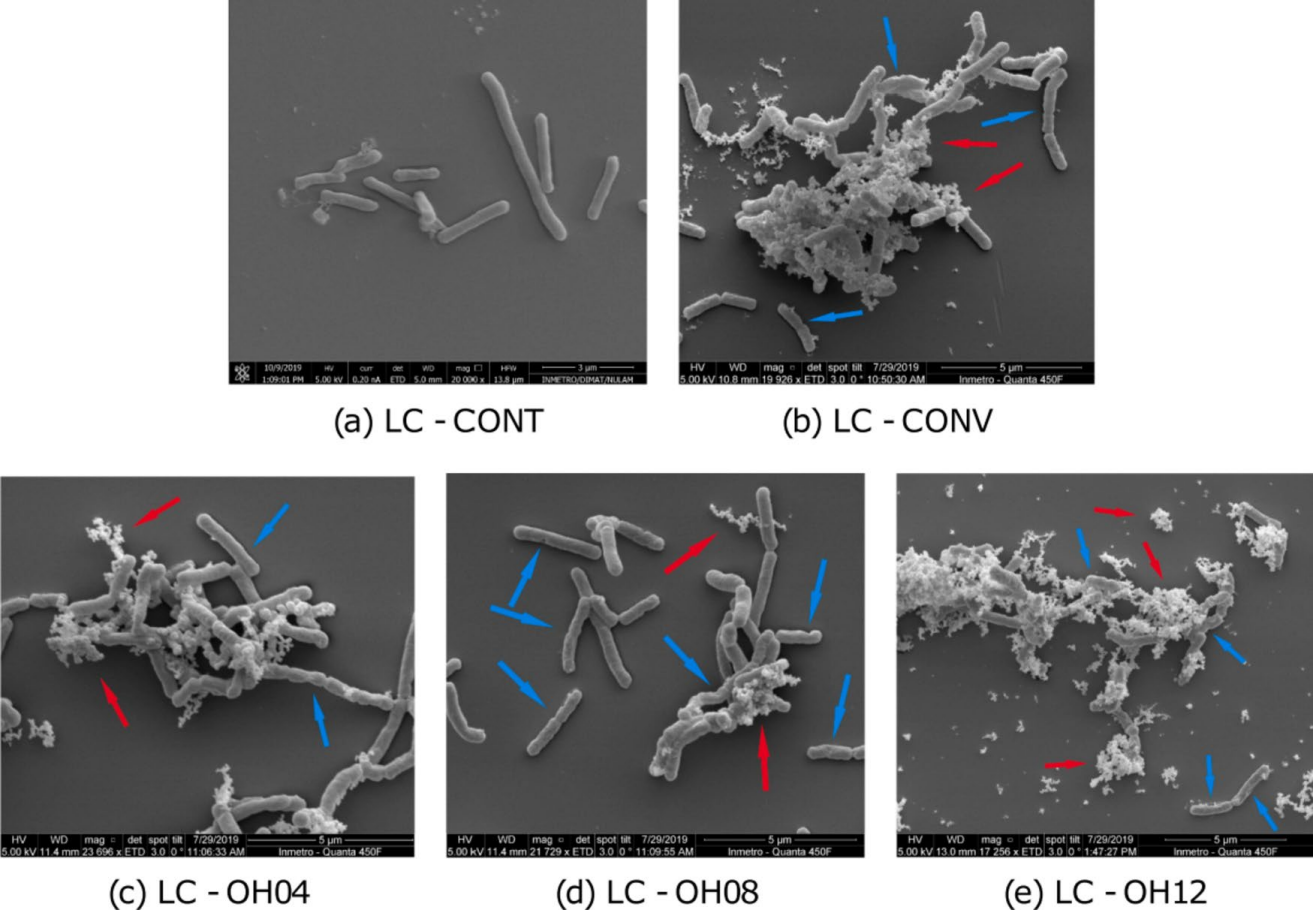
Scanning electron micrographs of *Lactobacillus casei* subsp. *paracasei* 01 (LC) **a** CONT, control; **b** cells after conventional heating (CONV); **c**, **d**, and **e** cells after ohmic heating at 4, 8, and 12 V/cm, 95 °C for 7 min, respectively. Blue arrows indicate damage and roughness on the cell surface while red arrows show ruptured cells and fragments/debris of the cell [20]

Inactivation via ohmic heating is achieved by passing an electrical current through the target microorganisms, causing them to heat up due to the resistance encountered by the electrical current. This process, also known as electrical resistance heating or Joule heating, leads to a rapid and even rise in the temperature of the substance which can potentially be used as an alternative technique to inactivate desired microbes for the production of probiotics as opposed to traditional heating methods. The inactivation mechanism of probiotics using ohmic heating is the same as that of traditional thermal treatments, along with non-thermal damages due to electroporation; however, ohmic heating has several advantages over conventional heating methods, including reduced processing time, enhanced quality retention of the food matrix, and improved energy efficiency [20, 45]. Electron micrographs of *Lactobacillus casei* subsp. *paracasei* after conventional and ohmic heating are shown in Fig. 4.

During the process of ohmic heating, the internal generation of heat ensures that substantial temperature gradients do not occur in the sample, thus reducing the risk of overheating. This stands in stark contrast to conventional methods, where uneven heat distribution within the sample is common. Nevertheless, this technique still faces challenges related to overheating or underheating in certain conditions. As the conductivity increases with temperature, controlling the endpoint temperature becomes difficult. Furthermore, the irregular shapes of treatment chambers can lead to local temperature variations, especially when dealing with diverse probiotic sample properties. Electrode geometries are customized to enhance uniform heating. Issues arise with the survival of microorganisms in crevices and temperature differences in mixtures of probiotic samples. Strategies such as using a heating medium with equal conductivity, combining ohmic heating with other methods, and developing specific models based on experimental data have been explored to address challenges and ensure precise temperature control during the preparation of paraprobiotics [46].

### Sonication

**Fig. 5:**
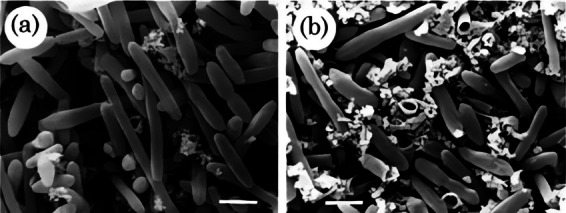
Scanning electron microscope (SEM) images of *L. delbrueckii* ssp. *bulgaricus* 11842 **a** before sonication treatment and **b** after sonication for 6 min [52]

Sonication is an alternative non-thermal method that employs ultrasound or sound waves with frequencies more than 20 kHz. The desired effects of sonication are caused by cavitation. The introduction of high-intensity ultrasound into a medium containing probiotics causes a cycle of alternating compression and rarefaction. This leads to the formation of bubbles or “voids”. These bubbles expand and collapse violently, generating high temperatures and pressures that can reach 5500 °C and 50,000 kPa, physically damaging the probiotic cells. The mechanical stresses and shock waves produced during cavitation can rupture cell membranes, disrupt cellular structures, and impair vital metabolic functions [47, 48]. Furthermore, sonication can be performed in combination with other treatments, such as UV and heat [24, 49]. The efficacy of ultrasonic inactivation is influenced by several factors, such as the ultrasound frequency and intensity, duration of exposure, target organism, and the properties of the liquid medium. The SEM images of *L. delbrueckii* ssp. *bulgaricus* after sonication treatment are provided in Fig. 5.

Different frequencies of ultrasound waves can be used to disrupt the cell wall and cell integrity of probiotics, thus facilitating the release of several biomolecules like proteins and DNA that have beneficial properties. Since the optimal frequencies depend on the strain used and the intended application, standardization is a critical step prior to inactivation in order to maximize the release of the necessary cellular component for the desired purpose. A study conducted on *Saccharomyces cerevisiae* showed the effect of different frequencies on the structure and growth of the microbe, with 20 kHz causing significant external damage to the cell morphology and structural integrity [50]. For probiotics, on the other hand, lower frequencies are used in order to maintain cell viability by reducing the sheer stress on the cells. Controlled sonication at low frequencies of up to 100 kHz enhances microbial growth and productivity; however, in certain cases, it can increase cell permeability. Further, studies have shown that the effect of sonication on microbes is also influenced by the amplitude intensity and duration of exposure. Another study conducted on *Lactobacillus brevis* demonstrated that sonication treatment at 23 kHz with an amplitude of 10 µm enhanced the cell count and proliferation rate while the same frequency caused cell death and hindered metabolic activity on increasing the amplitude to 15 µm [51]. Thus, probiotic-specific studies examining the effect of sonication at different intensities for different time periods are of utmost importance in fine-tuning the sonication parameters for the required cell viability, metabolite production, and biological function depending on the intended application.

### Ionizing Radiation (IR)

Other non-thermal methods can also be employed for inactivation of bacterial species. For instance, the irradiation method utilizes Ionizing Radiation (IR) like X-rays, electron beams, and gamma rays for microbial inactivation [53, 54]. There are no significant differences in the inactivation effectiveness of the three radiation sources [54, 55]. The primary sources of gamma rays are radioisotopes, such as Cobalt 60, which has a half-life of 5.27 years, and Cesium 137, which has a half-life of 30.17 years. The dose of irradiation applied is an essential factor to be considered in the irradiation process, which is measured in Grays (Gy). The radioresistance of bacterial species must be looked upon while opting for this process. In a study conducted by Sychev et al. [56], *Bifidobacterium bifidum* (5 × 10^8^ CFU/ml in powder form) was dissolved in water and irradiated with ^60^Co gamma rays, with doses in the range of 1 to 20 Gy, using an Issledovatel IN-1 irradiator. The optimal dose of gamma rays of 12 to 14 Gy was determined by measuring the concentration of superoxide dismutase, an antioxidant enzyme, in the medium. DNA is the principal cellular target that governs the loss of viability in this process; double-strand breaks are induced in the DNA due to the action of ionizing radiations. These radiations also generate reactive oxygen species (ROS), which further damage the DNA, eventually leading to cell death [56].

Apart from the radioresistance of the target organism, the dose of irradiation also depends on several other factors including the source of radiation, the penetration depth, the approved safety limits of irradiation, the cell components that need to be preserved, and the intended use of the paraprobiotic. An important parameter while deciding the dose is the decimal reduction dose or D_10_ value which is the irradiation dose that reduces the total cell viability to 10% of the original value when all other conditions including time, temperature, type of radiation, and microbial strain, are kept constant [57]. Gamma radiation is generally preferred for inactivation due to its established use in the preservation of food products such as vegetables, pulses, and fruits for human consumption [58]. A recent study published in 2022 by Porfiri et al. [57] using different strains of lactic acid bacteria demonstrated strain-specific differences in the probiotic responses to gamma irradiation. Strains showing higher resistance to gamma irradiation were seen to better preserve the beneficial properties of the live probiotics. The presence of a surface layer (S-layer) in the microbial cell wall of certain strains could potentially play a role in absorbing the radiation, thereby preserving the immunomodulatory cell wall components [57].

The effect of irradiation on paraprobiotics is examined post-treatment using different techniques. Cell viability is generally assessed through colorimetric assays like MTT and spectrofluorometric analyses [59]. The reproductive capability is evaluated via plate counting, colony-forming unit (CFU) assays, and growth curve analyses [60, 61]. Confocal laser scanning microscopy (CLSM) is generally employed to observe cell growth and assess structural integrity. Several approaches such as comet assays as well as polymerase chain reaction (PCR) methods like quantitative PCR and enterobacterial repetitive intergenic consensus PCR (ERIC-PCR) are employed to analyze DNA damage in inactive microbes [62]. Flow cytometry is an especially viable technique for the post-inactivation examination of paraprobiotics since several parameters including DNA content, cell size, and mitochondrial function can be measured parallelly by using specific fluorescent dyes. Table 2 contains a list of commonly used fluorescent dyes and the specific cell functions that they are used to assess [19].

**Table 2:** Main dyes used in flow cytometry and their functions. Reproduced with permission [19]

**Dye**	**Function(s)**	**References**
**TOTO-1** 1,19 (4,4,7,7-tetramethyl-4,7-diazaundecamethylene)-bis-4-[3-methyl-2,3-dihydro (benzo-1,3-oxazole)-2-methylidene]-1-(39-trimethylammonium propyl)-pyridinium tetraiodide	To assess membrane integrity	[63]
**DioC6 (3)** 3,3′-dihexyloxacarbocyanine iodide	To show respiration and membrane potential	[64]
**DiBAC4(3)** bis-(1,3-dibutylbarbituric acid) trimethine oxonol	To assess bacterial susceptibility to antibiotics and cell viability	[65]
**cFDA** Carboxyfluorescein diacetate	To evaluate cell esterase activity	[66]
**FDA** Fluorescein diacetate	To indicate enzyme activity of intracellular esterase	[67]
**HE** Hydroethidide	To measure reactive oxygen species (ROS)	[64]
**PI** Propidium iodide	To indicate cytoplasmic membrane integrity	[68]
**SYTO-9**	To assess membrane integrity	[69]

### Pulsed Electric Field (PEF)

The pulsed electric field is a non-thermal technology that can be used for the purpose of inactivating microorganisms. It is achieved by placing the target microbes between two electrodes that constitute a treatment chamber gap followed by the employment of short-duration pulses with high-voltage electric fields in the range of 5 to 90 kV/cm [70, 71]. The precise mechanisms underlying PEF-induced microbial inactivation are not fully explicated, but it has been shown that permeabilization of microbial membranes, i.e., electroporation, occurs due to the application of PEF. Under minimal pulsation conditions, the membrane damage would be reversible, whereas more severe conditions would lead to irreversible damage, ultimately resulting in cell death [70, 72, 73].

In order to optimize PEF parameters such as pulse duration, treatment duration, and electric field strength for achieving necessary inactivation levels, heat resistance mechanisms of the target microorganism must be studied, and the kinetics of PEF-induced inactivation must be understood. These kinetics can be assessed by measuring the microbial cell viability at varying strengths of the electric field with a uniform increase in the duration of treatment which is the product of the pulse width in µs and the total number of pulses. However, pulse application generates a Joule heating effect which, in turn, increases the electrical conductivity, thus altering the pulse width and electrical field strength [74]. Therefore, Heinz et al. [75] proposed the determination of optimal PEF parameters based on the total specific energy, which is influenced by the treatment duration, electrical field strength, and the treatment chamber’s electrical resistance properties. In the case of *Listeria monocytogenes*, the total specific energy and the treatment duration required for a specific inactivation level were seen to decrease upon increasing the electric field strength [74].

### Ultraviolet (UV) Rays

Ultraviolet (UV) rays are electromagnetic rays having wavelengths between 200 and 400 nm. They are non-ionizing rays that can be used for sterilization purposes. Damage to the DNA is considered the key lethal effect of UV on bacterial strains, the inactivation of probiotics results from the formation of pyrimidine dimers in DNA and RNA, which would lead to microbial death due to affected metabolic functions or mutations in key genes [19, 76]. Other lethal or sub-lethal damages include the formation of ROS, which react with DNA and cellular proteins [77]. Membrane permeability and molecular transport can also be affected by damage to the cell membrane which may lead to cell inactivation [78].

Generally, the process of UV inactivation includes exposing the live probiotics suspended in culture media to the UV source emitting light of a particular wavelength for a specified time period. The effectiveness of the treatment is then tested by plating the culture on an agar plate and checking for cell growth as well as colony formation after 72 h of incubation. The absence of detectable cell growth indicates that the inactivation procedure was successful. Currently, flow cytometry is a more commonly used technique to check for cell viability. In one study, *Lactobacillus rhamnosus* GG was inactivated by subjecting the cell suspension to UV radiation from a 39-W germicidal UV lamp placed at a distance of 10 cm for a duration of 5 min. Though the treated culture did not show any growth on the agar medium, it was able to reduce IL-8 levels in Caco-2 cells, as effectively as live *Lactobacillus rhamnosus* GG probiotics [79].

UV treatment is very effective in inactivating a variety of microorganisms while preserving their beneficial immunomodulatory properties. Studies have demonstrated negligible differences in the beneficial effects of certain microbes when administered in their live form, as probiotics, and in their UV-inactivated form, as paraprobiotics [22]. Being a non-thermal method, it does not damage cell wall components like peptidoglycans and lipoteichoic acid which interact with immune cells to regulate the immune system [80]. Since UV treatment does not use any toxic chemicals or reagents, there is no risk of harmful residues being left behind in the paraprobiotics. Additionally, UV treatment is currently used in several industries to treat products for human consumption like food and pharmaceuticals, making it a reliable and generally accepted method of inactivation. One common example is the UV-based sterilization of drinking water before its consumption and use for cooking purposes [81]. Furthermore, as thermal methods such as pasteurization may not kill spore-forming species, irradiation methods using UV and gamma rays provide a viable alternative. However, the effect of UV radiation on specific probiotic organisms remains unclear due to the influence of external parameters such as the germicidal wavelength, the duration of exposure, the target microorganism, and the specific strain used [78].

Techniques such as heat, ionizing radiation, and ultrasound can be combined with UV to produce a synergistic effect which would increase the efficiency of inactivation. In order to efficiently combine different treatments, the inactivation mechanisms involved in each technique must be understood [49, 82, 83].

Other inactivation methods include lyophilization, spray drying, and pH modification [19]. Lyophilization is achieved by freezing the target microorganisms, applying a high vacuum, and finally warming the microbes under a vacuum thus leading to the sublimation of water. In spray drying, the feed solution, containing probiotic cells and dissolved or suspended protectant solids, undergoes atomization to form small droplets that are rapidly dried in a hot airflow to convert them into particles [84]. The pH sensitivity of microorganisms can also be exploited to make them non-viable. As previously mentioned, the inactivation method used, its intensity and conditions, as well as the strain used may affect the properties of paraprobiotics. For instance, a study conducted by Ouwehand et al. [85] evaluated the inactivation of nine probiotic strains to assess the effect of heat, UV, and gamma irradiation on the adhesive property of probiotics using which they attach to the immobilized intestinal mucus. Inactivation by heat and gamma rays showed a decrease in the adhesive ability of the probiotics with the exception of *Propionibacterium freudenreichii* and *Lactobacillus casei* Shirota, respectively, where an opposite effect was observed. Inactivation by UV did not cause any difference in the adhesion properties.

Various inactivation parameters including intensity, duration, and frequency need to be tuned individually for specific applications. These parameters differ according to the strain used, microbial resistance patterns, initial microbial population, and environmental factors such as growth media. Hence, dose–response studies are necessary to optimize the treatment parameters and to choose a dosage that provides specific advantages.

## Gut-Brain Axis (GBA)

The gut microbiota has an essential role in human health and physiology, and gut dysbiosis, i.e., imbalance in the gut microbial populations, can have severe neuropsychiatric repercussions. Symptoms of anxiety and depression are explicitly correlated to alterations in the microbiota. Gut dysbiosis can occur due to various reasons such as eating habits, sleep patterns, diseased states, and medications such as antibiotics. Probiotics and paraprobiotics offer a viable solution for the restoration of a healthy, balanced gut microbiome [86, 87].

**Fig. 6:**
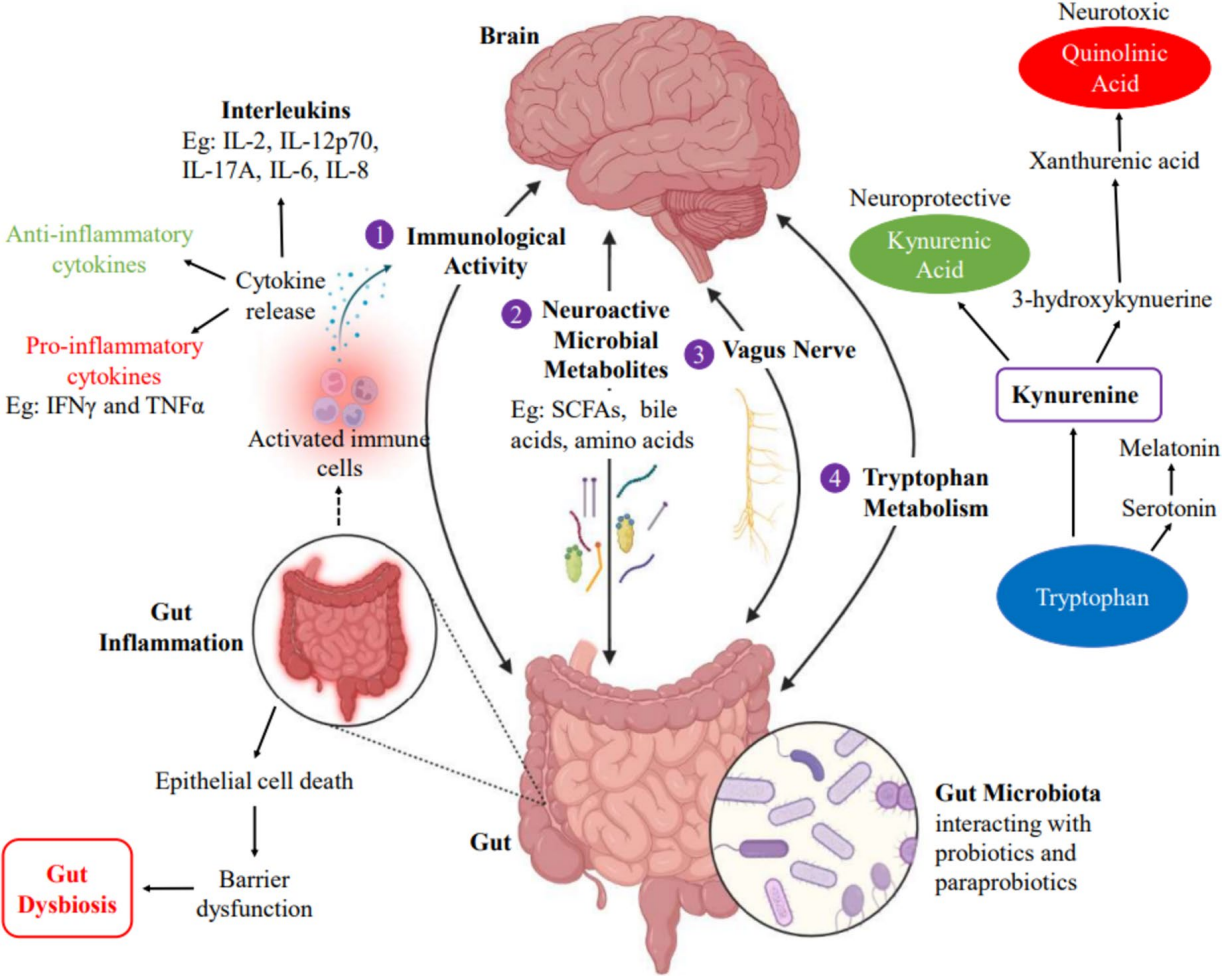
Pathways involved in the gut-brain axis (GBA)

This relationship between the gut microbes and the brain is mediated by a bidirectional communication termed the “Gut-Brain axis” that occurs through multiple different pathways which are regulated by various components as illustrated in Fig. 6.

### Vagus Nerve

**Fig. 7:**
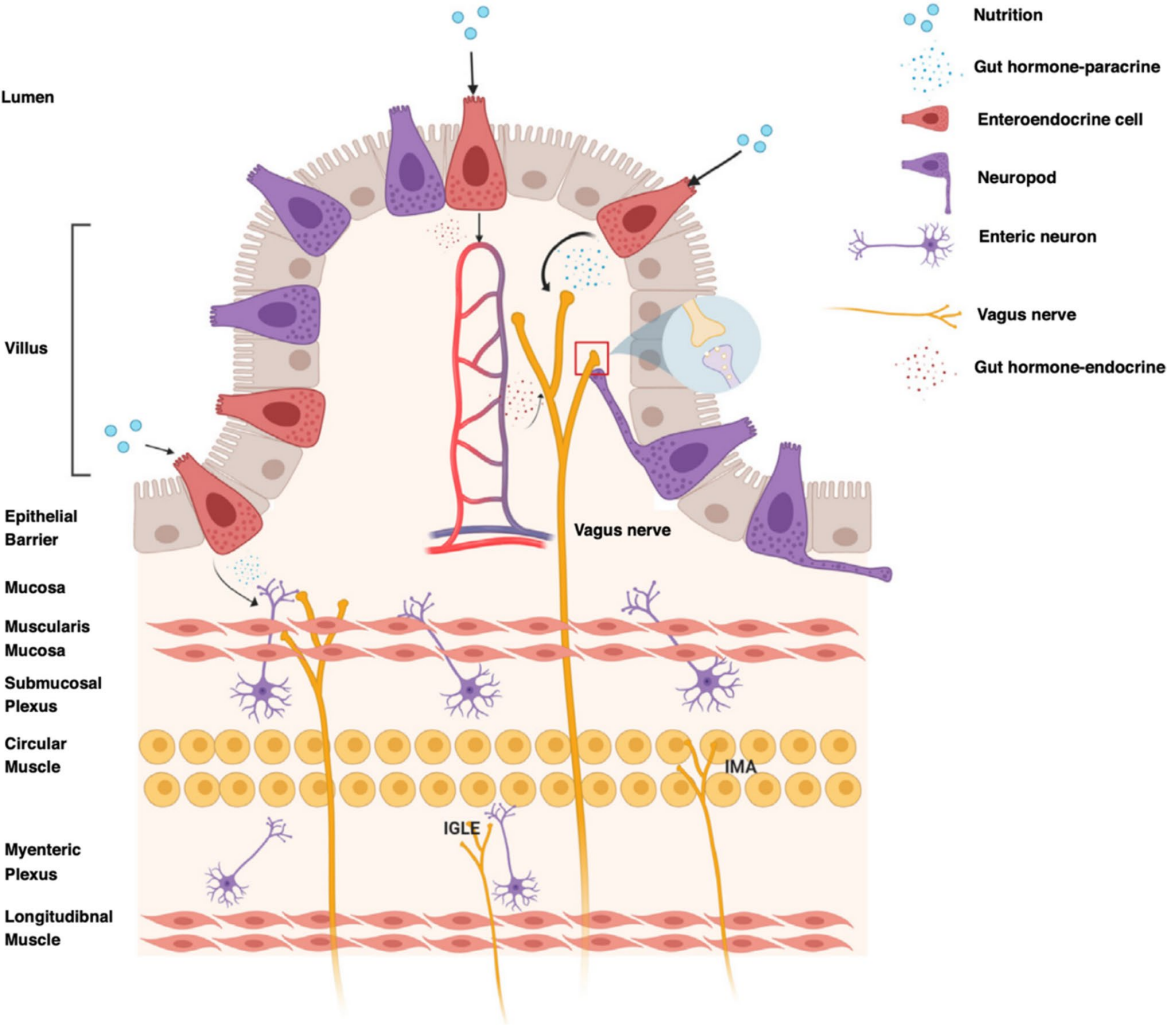
Diverse gut-brain connections through the vagus nerve [88]

The vagus nerve, the tenth cranial nerve in the autonomic nervous system, plays a critical role in gut-brain signaling [88]. It is a major constituent of the parasympathetic division and coordinates several physiological functions such as respiration, heart rate, and gut motility [89]. As demonstrated in Fig. 7, the sensory components of the vagus nerve transmit various sensory gut signals, including nutrient availability, microbial activity, and gut health, to the brain, thus affecting the host response [88]. This is primarily accomplished through vagal terminals formed along the gut epithelium by the vagal nerves which form two main detectors of mechanical signals—intra-ganglionic laminar endings (IGLEs) in combination with enteric neurons and intramuscular array endings (IMAs) in the muscle layers [90, 91]. The vagal afferent nerves that pass through the intestinal and antral glands as well as the taste buds in the proximal esophageal tract express specific receptors to detect serotonin, gastro-intestinal neurohormones, peptides, and other specific signaling molecules released by enteroendocrine cells in response to intestinal nutrient levels for the regulation of nutrient absorption and digestion. Special enteroendocrine cells that form synapses with vagal terminals are termed neuropods [88]. Enteroendocrine cells generally express receptors to detect fatty acids, glucose, and amino acids in the gut. Several microbial metabolites produced by the gut microbiota like lipopolysaccharides (LPS) are also detected by the enteroendocrine cells through toll-like receptors (TLRs) located on their surface [92]. Pathogenic microbes directly communicate with the vagus nerve through leaky gut barriers while beneficial microbes generally affect enteroendocrine signaling, potentially through a serotonin-dependent mechanism. These messages regarding food intake, digestion, gut microbial activity, nutrient absorption, and ultimately, gut health, are transmitted to specific regions of the brain, including the hypothalamus, the locus coeruleus, and the hippocampus through the nucleus tractus solitarius (NTS) which is the main processor of sensory signals from the gut [88]. Vagal efferent nerve fibers originate from the dorsal motor nucleus of the vagus in the brainstem and transmit motor signals from the brain to the enteric nervous system. The enteric neurons are triggered by vagal efferent nerves by the release of acetylcholine, thus regulating smooth muscle contractions that control peristalsis and food propulsion as well as the secretion of gastric acid, digestive enzymes, alkalis, mucus, and other substances necessary for digestion and nutrient absorption [93].

However, since neurotransmitters like serotonin, acetylcholine, and gamma-aminobutyric acid (GABA), as well as neuropeptides such as substance P, neuropeptide Y, and oxytocin, are produced by the endocrine nervous system, the vagal gut-brain signaling pathway also mediates stress response, mood, cognition, and overall brain function [94]. The excitation of the vagal afferent nerves in the gut has been associated with neuropsychiatric disorders like depression and anxiety [95]. In an experimental study, vagotomized mice were seen to be insensitive to probiotic treatment for anxiety-like behaviors, implying the imperative role of the vagus nerve in anxiolytic effects and gut-brain communication [96, 97]. Further, vagus nerve stimulation (VNS) therapy using electrical impulses was successful in reducing the symptoms of treatment-resistant depression (TRD). More specifically, the administration of the probiotic *Lactobacillus rhamnosus* (JB-1) led to increased levels of the neurotransmitter GABA in mice, which has implications for the treatment of anxiety and MDD [98].

### Immunological Activity

The immune system plays a crucial role in regulating the bidirectional interaction between the gut microbiota and the CNS, thus influencing the pathophysiology of neuropsychiatric disorders. Cytokines, produced by immune cells, are important messengers in the gut-immune-brain communication. They have two main types—proinflammatory cytokines that increase inflammation and anti-inflammatory cytokines that decrease inflammation. Gut bacteria can interfere with the balance between the two types of cytokines by upregulating the expression of one type of cytokines while downregulating the other. Increase in the expression of proinflammatory cytokines, such as IFNγ and TNFα, have been shown to cause central neuroinflammation, ultimately leading to psychological stress and depressive symptoms [99]. For instance, a study conducted on mouse models showed that the oral administration of heat-killed wild-type *Lactobacillus casei* as a paraprobiotic caused the suppression of IFNγ and TNFα. This immunomodulatory property can potentially be used in ameliorating the symptoms of neuropsychiatric disorders [100].

Interleukins are a subset of cytokines and specific interleukins are involved in the regulation of inflammation. During certain neuropsychiatric disorders like MDD, the NLRP3 inflammasome stimulates proinflammatory pathways by activating IL-1b signaling which is critical in the gut–immune–brain association [101]. Other proinflammatory interleukins include IL-2, IL-6, IL-8, IL-12p70, and IL-17A. As mentioned previously, it has been experimentally shown that UV-inactivated *Lactobacillus rhamnosus* GG (LGG) suppresses IL-8 production to the same extent as live LGG that is administered as a probiotic [79]. This introduces the possibility of using UV-inactivated LGG as an immunomodulatory paraprobiotic for certain psychological imbalances.

Microbial-associated molecular patterns (MAMPs) like flagellin and LPS are conserved, class-specific molecular structures present on the surface of microbes that are identified by pattern recognition receptors (PRRs) including Toll-like receptors (TLRs) and NOD-like receptors (NLRs) which are present on the surface of host cells and bind to specific MAMPs. However, since these MAMPs are produced by both pathogenic invaders and commensal gut microbes, several mechanisms are employed to prevent the destruction of the gut microbiota by the intestinal epithelial cells while also maintaining immune homeostasis. One particular mechanism is stratification, i.e., minimization of contact between gut microbes and intestinal epithelia which is accomplished by the mucus layers coating the inner wall of the intestine. Another strategy is compartmentalization in which gut microbiota are contained within specific zones to reduce their exposure to the systemic immune response. This is achieved by the epithelial production of antibacterial molecules like RegIIIγ which prevent gut microbes from reaching the small intestine as well as reduced gut microbial colonization in the stomach and duodenum due to their highly acidic environments [102, 103].

### Inflammatory Reflex and Neuroinflammation

The inflammatory reflex is a neurophysiological mechanism by which the vagus nerve mediates cytokine production, inflammation, and overall immune function. The afferent vagus nerve fibers regulate immune-to-brain communication by detecting peripheral inflammatory molecules and subsequently sending signals to specific regions of the brain, including the hypothalamus. The efferent vagus nerve is involved in cholinergic signaling from the brain to the immune system which suppresses cytokine production. Inflammatory mediators including bacterial peptides, lipopolysaccharides, and cytokines activate the afferent vagal nerves which ultimately leads to an increased production of the adrenocorticotropic hormone (ACTH) that has an anti-inflammatory effect. Afferent signals also hinder cytokine production by increasing glucocorticoid levels and stimulating the release of the melanocyte-stimulating hormone (MSH), an anti-inflammatory protein. Once these signals are transmitted to the brain, the efferent motor signals stimulate the release of acetylcholine (Ach), a neurotransmitter that binds to α7 nicotinic acetylcholine receptors (α7nAChR) on the surface of immune cells like macrophages, thus suppressing cytokine production [104–106]. A preclinical study observed increased inflammation due to high levels of TNF-α and IL-1β cytokines as well as depressive symptoms possibly due to BDNF-TrkB signaling by the brain-derived neurotrophic factor (BDNF) and its receptor, tropomycin receptor kinase B (TrkB) specifically in the nucleus accumbens region of the brain [107]. This is supported by experimental data that correlates inflammation-induced BDNF activity with the onset of depression in rat models [108].

Neuropsychiatric disorders like depression are known to be triggered by poor lifestyle and unhealthy diet which cause gut dysbiosis as well as stress which interferes with the hypothalamic–pituitary–adrenal (HPA) axis, thus eliciting a proinflammatory immune response [109]. The stress-induced immune response, coupled with the over-production of proinflammatory cytokines due to gut dysbiosis, can lead to neuroinflammation, i.e., inflammation in the central nervous system (CNS), through the gut-brain axis, which in turn dysregulates neurotransmitter metabolism, neural synaptic plasticity, and neuroendocrine function, ultimately affecting emotional regulation and overall behavior [110]. This is further supported by a clinical trial in which the use of 2 × 10^9^ CFU/g each of probiotics including *Bifidobacterium bifidum*, *Lactobacillus acidophilus*, and *Lactobacillus casei* was shown to have anti-depressive effects in MDD patients [111]. Microglia are immune effector cells that control neuroinflammation by releasing immune mediators and maintaining neuronal connectivity in the CNS. However, microglial activation by bacterial LPS and IFNγ could lead to the release of proinflammatory factors like IL-6, thus causing neurotoxicity due to increased neuroinflammation. Dysfunction of microglia has been associated with the onset of depression due to neuroinflammation in coordination with the disruption of the HPA axis [112, 113]. Studies have shown that specific microglial activation in certain parts of the brain like the prefrontal cortex plays a major role in MDD. A direct correlation has also been established between the severity of depression and the level of microglial activation in the anterior cingulate [114].

### Microbial Metabolites

Chemical compounds produced during microbe-mediated metabolic reactions are known as microbial metabolites. They include short-chain fatty acids (SCFAs), bile acids, phenols, thiols, and amino acids. Gut microbial metabolites affect the central nervous system, either directly or indirectly due to which gut dysbiosis can critically impact the pathophysiology of various CNS disorders, such as ASD. It has been observed that microbial metabolites can regulate ASD-like symptoms both positively and negatively. A decrease in levels of SCFAs like butyrate has been noted in the fecal samples obtained from ASD patients [115]. It has been experimentally shown using a mouse model that the administration of C_4_ fatty acids like sodium butyrate can attenuate ASD symptoms and improve social behavior by regulating the expression of ASD-related genes [116].

SCFAs are organic compounds formed as end-products of microbial fermentation, especially in the caecum and proximal colon of the gastrointestinal tract. The major SCFAs in the gut include propionate, acetate, and butyrate. SCFAs help in the maintenance of gut barrier integrity via the regulation of tight-junction proteins and the stimulation of increased production of mucin, a constituent of the mucus lining, by the gut epithelium [117]. In vitro studies have demonstrated the ability of SCFAs to improve intestinal permeability and epithelial barrier function in a concentration-dependent manner [118, 119]. SCFAs mediate immune homeostasis in the gut by regulating inflammation. Preclinical trials have shown the ability of SCFAs to upregulate the production of IL-10-producing regulatory T-cells, thus increasing the levels of IL-10, an anti-inflammatory cytokine [120]. They also interact with the gut-brain-axis by binding to G-protein coupled receptors (GPCRs) on the surface of enteroendocrine cells and triggering the production of neuropeptides like glucagon-like peptide 1 (GLP-1), ghrelin, and peptide YY [121]. The role of SCFAs in energy metabolism is primarily through butyrate oxidation which accounts for up to 70% of the energy supply to colonocytes [122]. SCFAs play a role in regulating the metabolism of glucose and lipids by binding to specific GPCRs. They activate the oxidation of fatty acids while inhibiting fatty acid synthesis and lipolysis through an AMPK-mediated pathway [123]. Moreover, SCFAs also influence the production of neurotransmitters in the brain. Acetate has been shown to increase the hypothalamic levels of GABA and lactate in vivo, possibly via anorectic signaling in the arcuate nucleus [124]. In vitro studies have demonstrated that SCFAs like butyrate and acetate modulate serotonin levels by regulating the expression of *tph1* which encodes tryptophan 5-hydroxylase 1, the enzyme necessary for serotonin production [125]. Similarly, propionic acid and butyric acid regulate the expression of tyrosine hydroxylase which is required for the production of major neurotransmitters such as epinephrine, norepinephrine, and dopamine [121, 126]. Thus, the use of microbial metabolites, especially particular types of SCFAs, as paraprobiotics in disorders like ASD is currently being explored, but their clinical application remains challenging due to the heterogeneity of the results.

### Neuroactive Compounds

Neuroactive compounds include a variety of substances like neurohormones, neuropeptides, neurohormones, neuromodulators, mediators, and metabolites from various sources that influence the neural system and thereby affect brain function as well as other CNS-related activities. Since they play a vital role in mediating and regulating the microbiota-gut-brain signaling pathways, abnormal levels of these compounds are associated with the pathogenesis of neuropsychiatric disorders such as ASD and MDD [127].

Tryptophan metabolism is an important component of the GBA. The metabolism of tryptophan, a derivative of indole, to kynurenine, can be either upregulated or downregulated depending on the gut microbes involved. Kynurenine can either be metabolized into kynurenic acid which is a neuroprotective metabolite or into quinolinic acid. It has been experimentally observed that MDD patients have lower kynurenic acid levels even though tryptophan metabolism occurs at a fast rate, possibly due to the preferential conversion of tryptophan into quinolinic acid [128]. This reduction in kynurenic acid levels causes a disruption in the neuroprotective and neurodegenerative cascades, thus contributing to the development of depressive symptoms. Thus, in order to manage the pathophysiology of MDD, kynurenine levels must be controlled. Experimental evidence indicates that treatment with *Bifidobacterium* spp., in combination with an improved diet, can reduce kynurenine levels and improve depressive symptoms due to which it is a promising probiotic that can be used to manage symptoms in MDD patients [129].

Additionally, several neuroactive indole derivatives like serotonin, indole-3-propionic acid (IPA), and melatonin are produced by the gut microbiota during tryptophan metabolism. The overproduction of indole has been linked to symptoms of anxiety and depression in preclinical trials, thus implicating individuals with gut microbiota that produce higher levels of indole in the development of neuropsychiatric problems [130]. Indole alkaloids influence neural transmission by interacting with GABA receptors and serotonin receptors, thus exhibiting an anti-depressive effect that can potentially be used in neuropsychiatric therapy [131]. Further, indole derivatives like IPA and melatonin have been shown to act as neuroprotectants via their antioxidant and anti-inflammatory activities, thus regulating neural signaling [132]. Indole derivatives exhibit these neuroprotective effects both in vitro and in vivo by increasing dopamine levels and enhancing dopamine uptake, scavenging ROS to alleviate oxidative stress, and modulating cytokine production, including the downregulation of TNF-α, IL-1β, and IL-6, in order to control neuroinflammation [133].

## Applications

Several studies have established the viability of probiotics and paraprobiotics in the management of neuropsychiatric disorders including MDD, ASD, and anxiety.

Clinical and preclinical studies indicating the health benefits of probiotics are summarized in Table 3.

**Table 3:** Effects of probiotics on neuropsychiatric symptoms and disorders

**Probiotic**	**Subject(s) and type**		**Dose and duration**	**Observed neuropsychiatric effects**	**References**
***Lactobacillus casei* Shirota**	Clinical trial	General population (*n* = 63)	6.5 × 10^9^/65 mL of Yakult milk-based drink	Improved mood in depressive subjects	[134]
***Lactobacillus helveticus* R0052** ***Bifidobacterium longum* R0175**	Preclinical study	Male Wistar rats (*n* = 12)	10^9^ CFU/dose of 5 mL/kg via oral gavage for 2 weeks	Anxiolytic effects in rats and amelioration of depressive symptoms in humans	[135]
	Clinical trial	Healthy Caucasian men and women (*n* = 26)	3 × 10^9^ CFU in a probiotic stick for 30 days		
***Lactobacillus rhamnosus* R0011** ***Lactobacillus helveticus* R0052**	Preclinical study	MS rats	1 × 10^9^ CFU/mL via water	Mitigation of the negative effects of stress on the next generation in rats	[136]
***Lactobacillus helveticus* R0052** ***Bifidobacterium longum* R0175**	Clinical trial	Patients with low to moderate depression (*n* = 28)	≥ 10 × 10^9^ CFU/ sachet taken orally for 8 weeks	Antidepressant activity possibly by an increase in BDNF levels	[137]
***Lactobacillus acidophilus Lactobacillus fermentum*** ***Bifidobacterium lactis*** ***Bifidobacterium longum***	Preclinical study	Male Wistar rats (*n* = 12)	1 × 10^10^ CFU/2 g of probiotic powder in 30 mL of water	Improvement in oxidative stress biomarkers, enhanced cognitive function, and amelioration of memory and learning impairments in Aβ_1-42_-injected rats	[138]
***Streptococcus thermophilus*** ***Bifidobacterium longum*** ***Bifidobacterium breve*** ***Bifidobacterium infantis*** ***Lactobacillus acidophilus*** ***Lactobacillus plantarum*** ***Lactobacillus paracasei*** ***Lactobacillus delbrueckii* subsp. *bulgaricus*** ***Lactobacillus brevis***	Preclinical study	3 × Tg-AD mice (*n* = 32)	200 billion bacteria/kg/day for 4 months	Reduction in brain damage, decrease in Aβ_1–42_ and proinflammatory cytokines, and increase in SCFAs and hormones with neuroprotective properties such as leptin, GLP-1, ghrelin, and GIP	[139]
***Bifidobacterium longus*** ***Lactobacillus acidophilus***	Preclinical study	APP/PS1^TG^ mice (*n* = 8)	120 mg/day, five times a week for 20 weeks	Reduction in Aβ plaques and enhanced cognitive performance in MWM test	[140]
***Lactobacillus acidophilus Lactobacillus casei Bifidobacterium bifidum*** ***Lactobacillus fermentum***	Clinical trial	AD patients (*n* = 30)	2 × 10^9^ CFU/g each in a 200 mL probiotic drink for 12 weeks	Patients treated with probiotics demonstrated a notable improvement in the MMSE score	[141]
***Bifidobacterium breve* A1**	Clinical trial	Suspected MCI patients (*n* = 40)	≥ 1 × 10^10^ CFU per capsule for 16 weeks	Improvement in RBANS score as compared to the placebo group, indicating improved memory function	[142]
***Bifidobacterium longum Bifidobacterium bifidum Bifidobacterium lactis*** ***Lactobacillus acidophilus***	Clinical trial	Patients with GAD (*n* = 16)	1.8 × 10^10^ CFU in a capsule for 8 weeks	The combination of probiotics and sertraline (PS) was more effective than using sertraline (S) alone in reducing symptoms of anxiety in patients diagnosed with GAD (as reflected by the larger decrease in HAM-A scores in the PS group)	[143]
***Lactobacillus acidophilus*** ***Lactobacillus casei*** ***Bifidobacterium bifidum***	Clinical trial	Patients with MDD (*n* = 20)	2 × 10^9^ CFU/g each in a capsule for 8 weeks	Probiotic supplementation for 8 weeks in patients suffering from MDD had positive effects on BDI scores	[111]
***Lactobacillus helveticus* IDCC3801**	Clinical trial	Healthy older adults (*n* = 26)	125, 250, and 500 mg of tablets four times a day for 12 weeks	Improvement in cognitive tests (such as DST, SRT, and VLT) as compared to the placebo group	[144]
***Lactobacillus helveticus Bifidobacterium longum***	Clinical trial	Depressed patients (*n* = 38)	≥ 10 × 10^10^ CFU per 5 g sachet for 8 weeks	An enhanced BDI score compared to the placebo group was observed	[145]
***Lactobacillus rhamnosus* (JB-1)**	Preclinical study	Adult male BALB/c mice (*n* = 16)	1 × 10^9^ via oral gavage for 28 days	*L. rhamnosus* alleviated anxiety and depression-related behavior in mice via the vagus nerve and altered GABA receptor expression	[97]
***Lactobacillus casei* Shirota**	Clinical trial	CFS patients (*n* = 19)	8 billion CFU thrice daily for 8 weeks	Probiotics ameliorated symptoms of anxiety vs controls in subjects suffering from chronic fatigue syndrome	[146]
***Bifidobacterium longum* NCC3001**	Clinical trial	Patients with mild to moderate anxiety and/or depression and IBS (*n* = 18)	1 × 10^10^ CFU/g powder in lactose-free milk for 6 weeks	At weeks 6 and 10, patients under the probiotic group showed anti-depressant activity but not anxiolytic properties (assessed by the HAD scale); the probiotic group also showed alterations in brain activity as observed through fMRI	[147]
***Bifidobacterium bifidum*** ***Bifidobacterium lactis*** ***Lactobacillus acidophilus Lactobacillus brevis*** ***Lactobacillus casei*** ***Lactobacillus salivarius*** ***Lactococcus lactis***	Clinical trial	Young adults (*n* = 20)	> 2.5 × 10^9^ CFU/g in a 2 g powder for 4 weeks	After 4 weeks, subjects receiving probiotics experienced a significant decrease in cognitive response to sad moods	[148]
***Lactobacillus gasseri* CP2305**	Clinical trial	Male medical students enrolled in a cadaver dissection course (*n* = 24)	1.0 × 10^10^ CFU in a 2.5 g powder for 4 weeks	Improvement in anxiety, depressive states, and sleep quality	[149]
***Lactobacillus casei***	Preclinical study	MS to establish PPD in Sprague Dawley rats (*n* = 8)	8 × 10^8^ CFU/kg/day from postnatal day 2 to day 28	Improvement in depressive symptoms induced by MS via the optimization of gut microflora, amelioration of oxidative stress, and BDNF-ERK1/2 signaling activation	[150]

*n* number of intervention groups, *BDNF* brain-derived neurotrophic factor, *Aβ* amyloid beta, *3* × *Tg* triple transgenic mice used as an Alzheimer’s disease model, *AD* Alzheimer’s disease, *SCFA* short-chain fatty acid, *GLP-1* glucagon-like peptide 1, *GIP* glucose-dependent insulinotropic polypeptide, *MWM* Morris water maze, *MMSE* mini-mental state examination, *MCI* mild cognitive impairment, *RBANS* repeatable battery for the assessment of neuropsychological status, *GAD* generalized anxiety disorder, *HAM-A* Hamilton rating scale for anxiety, *MDD* major depressive disorder, *BDI* Beck depression inventory, *GABA* gamma-aminobutyric acid, *fMRI* functional magnetic resonance imaging, *CFU* colony-forming units, *MS* maternal separation, *DST* digit-span test, *SRT* story recall test, *VLT* verbal-learning test, *CFS* chronic fatigue syndrome, *IBS* irritable bowel syndrome *HAD* hospital anxiety and depression scale, *PPD* postpartum depression

Though research is still in the nascent stage as compared to probiotics, several preclinical and clinical studies have demonstrated the favorable neuropsychiatric effects of different paraprobiotics as well. The studies, along with their results, have been encapsulated in Table 4. Unlike probiotics, paraprobiotics cannot multiply, making the dose size an important consideration. In one particular study, Murata et al. [151] employed 2 different dosage forms—1 × 10^10^ (10 LP) and 3 × 10^10^ (30 LP)—on human subjects. The study found that the supplementation had a greater positive impact on both common cold and mood in the 10 LP group compared to the 30 LP group. The researchers believe that this may be due to the dose-independent nature of the treatment since probiotics generally exert immunomodulatory effects in a dose-dependent manner [152]. However, this difference in positive impact may also be due to the fact that the 30-LP group had a smaller sample size, as more participants dropped out before the intervention in this group. In contrast, another study testing the efficacy of *Bacillus* sp. NP5 paraprobiotic at different density levels on the resistance of Nile tilapia fish to *Streptococcus agalactiae* infection showed that maximum immune response and disease resistance was observed at the highest dose of 10^10^ CFU/mL [153]. The dependency of health benefits on the dosage size is yet to be conclusively determined since many studies assessing the effect of paraprobiotics on neuropsychiatric symptoms have not considered possible changes in the observed effects upon altering the dosage while keeping all other factors constant. Additionally, comparative trials between probiotics and paraprobiotics at different concentrations on the same subjects help to elucidate the required change in dosage upon inactivation. For instance, a recent study conducted by Elham et al. [154] revealed that the cytotoxic effect of live *L. casei* probiotic on CaCo2 cells was much higher than that of the heat-inactivated paraprobiotic from the same microbial strain at each concentration that was tested, indicating that the inactivated paraprobiotic must be administered in higher doses to elicit a similar effect as compared to the viable probiotic. Further, both treatments showed a dose-dependent increase in their cytotoxic potential. It is important to note, however, that these results are specific to the microbial species and cell line in question and cannot be generalized for all probiotics and paraprobiotics. Research into this particular aspect in the context of neuropsychiatric disorders is critical to determine whether a similar increase in dosage will be required for inactivating probiotics that have the ability to alleviate neuropsychiatric symptoms.

**Table 4:** Inactivation methods, dosage, duration, and neuropsychiatric effects of paraprobiotics

**Paraprobiotic**	**Subject(s) and type**		**Inactivation method and conditions used**	**Dose and duration**	**Observed neuropsychiatric effects**	**References**
***Lactobacillus paracasei*** **PS23**	Preclinical study	CS-treated male C57BL/6 J mice (*n* = 8)	Heat-killed at 100 °C for 30 min	10^8^ cells/0.2 mL daily via oral gavage for 41 days	▪ The paraprobiotic reversed the reduced dopamine levels due to CS ▪ Reduction in anxiety and depression-like behaviors via the hippocampal expression of BDNF, MR, and GR was observed	[155]
***Lactobacillus paracasei*** **PS23**	Preclinical study	MS Mice (*n* = 8)	Heat-killed at 100 °C for 25 min	1 × 10^9^ cells via oral gavage for 4 weeks	▪ Mitigation of stress, anxiety, and depressive behaviors in MS mice ▪ Higher levels of IL-10 and lower serum corticosterone levels were also observed	[156]
***Lactobacillus gasseri*** **CP2305**	Clinical trial	Medical students undertaking an examination (*n* = 34)	Pasteurized at 90 °C and freeze-dried	1 × 10^10^cells/200 mL container via a fermented milk beverage for 12 weeks	▪ Decreased salivary cortisol levels which are directly linked to the levels of psychological distress ▪ Improved sleep quality as confirmed by EEG ▪ Increased parasympathetic nerve activity and better bowel movement	[157]
***Lactobacillus gasseri*** **CP2305**	Clinical trial	Healthy young women (*n* = 25)	Pasteurized at 90 °C and freeze-dried	1 × 10^10^ bacterial cells per 2 tablets	▪ Amelioration of anxiety, depressive mood, or related psychological premenstrual syndrome	[158]
***Lactobacillus gasseri*** **CP2305**	Clinical trial	Male ekiden runners (*n* = 24)	Pasteurized at 90 °C and freeze-dried	1 × 10^10^/ 200 mL of isotonic sports beverage for 12 weeks	▪ The consumption of CP2305 proved to be beneficial in recovering from fatigue and alleviating feelings of anxiety and depression throughout the demanding training phase	[159]
***Enterococcus faecalis*** **EC-12**	Preclinical study	C57BL/6 J mice (*n* = 8)	Heat-killed	AIN-93 M diet with EC-12 concentration of 0.125% for 4 weeks	▪ Attenuated anxiety and depression-like symptoms as assessed by OFT, EPM, and FST ▪ Enhanced expression of neurotransmitter receptor genes such as *Adrb3* and *Avpr1a* in the prefrontal cortex (*Avpr1a* has been shown to influence socio-emotional behavior, and polymorphisms in *Avpr1a* have been associated with a predisposition to social impairments in ASD)	[150, 160]
***Lactobacillus paracasei*** **MCC1849**	Clinical trial	Healthy adults (*n* = 132)	Heat-killed	1 × 10^10^ cells (*n* = 69) and 3 × 10^10^ (*n* = 63) cells orally in a milk powder	▪ Supplementation had the ability to boost resistance to common cold infection while also maintaining a healthy mood state even under stressful conditions	[151]

*n* number of intervention groups, *BDNF* brain-derived neurotrophic factor, *MR* mineralocorticoid receptors, *GR* glucocorticoid receptors, *CS* corticosterone, MS maternal separation, *IL-10* interleukin-10 (anti-inflammatory cytokine), *EEG* electroencephalogram, *OFT* open-field test, *EPM* elevated plus-maze test, *FST* forced swim test, *ASD* autism spectrum disorder

## Conclusion

There exists a paradoxical relationship between neuropsychiatric disorders and gut dysbiosis. The GBA mediates a two-way communication between the gut microbiota and the brain, thus facilitating the involvement of the gut microbes in the development and regulation of the nervous system. Thus, gut dysbiosis plays a major role in the pathogenesis of neuropsychiatric disorders such as ASD and MDD. Evidence suggests that both probiotics and paraprobiotics possess the ability to ameliorate neuropsychiatric symptoms and disorders through the GBA due to their potential to optimize the gut microbiome through interactions with the gut microbes. However, probiotics pose several safety concerns including susceptibility of immunocompromised patients and development of antibiotic resistance. Despite the advantages of paraprobiotics over probiotics in terms of safety and shelf life, they are not without limitations. The main disadvantage of paraprobiotics is the lack of standardized inactivation protocols which are necessary for their commercial production and quality control. Further research is required to compare and establish standard parameters for paraprobiotic production since the inactivation of probiotics by different methods may lead to variations in the properties of the paraprobiotics obtained and their conferred health benefits. Moreover, the mechanism of action of paraprobiotics and the role of specific cellular components must be elucidated. Research into this aspect is hindered by the difficulty in accurately determining the cell viability of probiotics, resulting in the erroneous attribution of the beneficial effects of paraprobiotics to their viable counterpart. Additionally, specific criteria must be established for choosing the target microorganisms, and definitive tests are required for determining the biological activity of inactivated microbes. Most importantly, the efficacy and sustainability of paraprobiotics in the gut must be assessed, given their inability to reproduce. Reports suggest that gut mucosal colonization resistance to probiotics may nullify their potential health benefits [161]. Therefore, time-point assays need to be performed to determine the optimal treatment duration and the prolongation of health benefits after the treatment period. In order to translate the potential advantages of paraprobiotics into health benefits, the optimum dosage, frequency and the total duration of consumption need to be evaluated [162]. Unless paraprobiotics are able to induce lasting changes in the gut microbiota, their health benefits will be limited to the time period of administration, unlike probiotics which proliferate and colonize in the gut, leading to long-term beneficial effects. While paraprobiotics may be able to rectify gut dysbiosis by regulating the growth of different microbial populations thereby producing long-term health benefits, it is difficult to reach a definitive conclusion due to the lack of sufficient experimental evidence, thus highlighting the exigent need for further research. In conclusion, though further work is necessary to optimally channel their beneficial properties, probiotics and paraprobiotics are highly viable natural therapeutic agents for the management of neuropsychiatric disorders.

## Data Availability

Data sharing is not applicable to this review article as no datasets were generated or analyzed during the current study.
